# Multicomponent exercise interventions for cognitive function in patients with mild cognitive impairment: a summary of best evidence

**DOI:** 10.3389/fresc.2025.1696284

**Published:** 2025-12-19

**Authors:** Zhitong Zhang, Jia Wang, Xinhua Qiao, Shan Fu, Liying Ma, Yanli Wang

**Affiliations:** 1 School of Nursing, Inner Mongolia Medical University, Hohhot, China; 2 Department of Gynaecology, Inner Mongolia People’s Hospital, Hohhot, China; 3 Cardiovascular Combined Unit, Inner Mongolia People’s Hospital, Hohhot, China; 4 Executive Health Ward, Inner Mongolia People’s Hospital, Hohhot, China; 5 Department of Nursing, Inner Mongolia People’s Hospital, Hohhot, China

**Keywords:** aged, mild cognitive impairment, cognitive function, multicomponent training, evidence summary, evidence-based nursing

## Abstract

**Aim:**

This review systematically analyzes and synthesizes evidence on multicomponent training interventions aimed at improving cognitive function in older adults with mild cognitive impairment (MCI). The goal is to inform clinical practice with actionable insights.

**Design:**

Best evidence summary.

**Methods:**

This review adhered to the 6S model to identify evidence, searching data sources from their inception to April 30, 2025. The process involved systematic screening, quality appraisal, and data extraction for evidence synthesis.

**Results:**

A total of 12 sources were included: one guideline, six systematic reviews, one expert consensus, three evidence summaries, and one meta-analysis. From these sources, 24 best-evidence statements were synthesized across six domains: (a) principles for developing multicomponent training prescriptions, (b) intervention effects, (c) exercise dosage and intensity, (d) safety monitoring and risk management, (e) outcome assessment methods, and (f) strategies for promoting implementation and adherence.

**Conclusion:**

The evidence suggests that healthcare professionals should tailor multicomponent training interventions to the individual needs and clinical contexts of older adults with MCI to effectively slow cognitive decline.

**Relevance to clinical practice:**

This synthesis provides an evidence-based framework for developing multicomponent training protocols for older adults with MCI, supporting targeted interventions that may mitigate cognitive deterioration.

**Summary:**

This evidence synthesis integrates 12 high-quality sources, generating 24 best practice recommendations across six critical domains, including prescription design, exercise dosage, and safety management, offering guidance for personalized clinical application.

**Systematic Review Registration:**

Identifier [ES20257822].

## 1 Introduction

Mild cognitive impairment (MCI) is a clinical condition characterized by cognitive dysfunction that lies between normal aging and dementia, and it may be reversible (1). As the global population continues to age, it is projected that by 2025, individuals aged 60 and older will account for over 14% of the global population. In China, by 2023, the proportion of individuals aged 60 and above had already reached 21.1% (2, 3). This growing older adult demographic has led to a significant increase in the number of individuals diagnosed with MCI and Alzheimer's disease (AD) (4). Recent surveys indicate that the prevalence of MCI among the older adult in China is approximately 15.5%, affecting around 38.87 million individuals, many of whom are at high risk of developing more severe cognitive disorders (5). The high prevalence of MCI and its potential progression to dementia underscore the importance of early diagnosis and intervention during this critical period. Patients with MCI exhibit cognitive decline that exceeds what is expected for their age and educational level, particularly in areas such as memory, attention, executive function, and language (6). Unlike patients with dementia, individuals with MCI have not yet met the criteria for dementia and still retain substantial cognitive reserve and plasticity. This preserved cognitive function offers a unique opportunity for early intervention to prevent further deterioration.

Currently, drug therapy remains the primary clinical approach for managing MCI, particularly the use of cholinesterase inhibitors. However, evidence regarding the long-term cognitive benefits of these medications remains inconclusive, and they are associated with various side effects. The American Academy of Neurology (AAN) has explicitly stated that cholinesterase inhibitors should not be routinely prescribed for the treatment of MCI (7). In contrast, non-pharmacological interventions are gaining attention due to their favorable safety profile and accessibility. Among these, exercise interventions have shown promise in improving cognitive function in MCI patients (8). Previous studies have focused on single-component exercise programs, such as aerobic or resistance training, which are easy to standardize and have been foundational in improving cognitive function in MCI patients (9). However, recent studies suggest that individuals with MCI may benefit more from complex exercise programs involving multi-task coordination (10). These complex exercises have the potential to reorganize neuronal firing patterns in higher-order cortical regions, such as the prefrontal cortex, thereby enhancing executive control, decision-making, and attention regulation (11). For example, incorporating cognitive tasks, such as mental arithmetic or memory exercises, during physical activities like walking can stimulate both sensory-motor and cognitive processing regions in the brain, promoting neuroplasticity (12). Such frequent task-switching training has been shown to significantly improve executive control functions in MCI patients.

Multicomponent training (MT) is an integrated exercise regimen that combines various forms of physical activity, including flexibility, balance, and coordination exercises, alongside conventional aerobic and resistance training. Its defining characteristic is the incorporation of multiple exercise modalities. In the context of cognitive function interventions, MT can be effectively combined with cognitive training. One powerful approach to integration is through a dual-task paradigm (e.g., performing cognitive tasks such as calculations while exercising), which simultaneously challenges both physical and cognitive systems (13). It is important to emphasize that while dual-task training is a common and effective strategy within MT-based cognitive interventions, the two concepts are distinct: MT refers to the combination of different physical exercises, whereas dual-task training is a delivery mode that can be applied within various exercise formats. This integrated approach enhances neural plasticity by stimulating multiple systems and challenging cognitive reserves, potentially leading to more significant improvements in cognitive function. MT has increasingly been recognized as one of the most effective exercise regimens for older adults (14).

Despite the promising potential of multicomponent training (MT), its application in older adult patients with mild cognitive impairment (MCI) is hindered by three key issues: (1) significant heterogeneity in intervention protocols, including variations in exercise modalities, intensity, frequency, duration, and the lack of standardized protocols (15, 16); (2) unclear evidence quality and inconsistent recommendations, as existing guidelines and systematic reviews lack evidence-based recommendation grading (17); and (3) inadequate consideration of clinical applicability, with many intervention designs failing to account for patients' functional status, preferences, and practical feasibility in medical or community settings (18). This study aims to address these gaps by following the Joanna Briggs Institute (JBI) evidence-based healthcare model, conducting systematic literature searches, and employing rigorous evaluation tools such as AGREE II, CASP, and GRADE to develop evidence-based MT practice guidelines. These guidelines will be tailored to patient preferences and clinical contexts, with the goal of enhancing precision-based exercise interventions for older adult individuals with MCI. In summary, this study will utilize evidence-based medicine methods to systematically review and integrate the best available evidence, providing standardized and actionable guidance for clinical practice in MCI management.

## 2 Methods

### 2.1 Evidence-based question formulation

To address the clinical question, “How does multicomponent training (MT) affect the cognitive function of older adult patients with mild cognitive impairment (MCI)?” the primary research inquiry was structured using the PICOTS evidence-based framework (19):

P (Population): older adult individuals diagnosed with mild cognitive impairment;

I (Intervention): Multicomponent training, including endurance, strength, balance, and flexibility exercises;

C (Comparison): Alternative non-MT interventions, such as single-component exercise or health education;

O (Outcome): Cognitive function, assessed using standardized instruments such as the Mini-Mental State Examination (MMSE) or the Montreal Cognitive Assessment (MoCA);

T (Timing): A minimum intervention duration of 3 months, with optimal effects expected at 6 months or longer. Outcome assessments are planned at baseline (T0), 4–8 weeks post-intervention (T1), at the conclusion of the intervention (T2), and at 3 months (T3) and 6 months (T4) post-intervention (13);

S (Setting): Various settings, including hospital departments (geriatrics, neurology, rehabilitation), community environments (e.g., health service centers, senior activity centers), and long-term older adult care facilities.

This project has been registered with the Fudan Evidence-Based Nursing Center under registration number ES20257822.

### 2.2 Search Strategy

A comprehensive literature search was conducted following the top-down “6S” model of evidence retrieval (20), with the search updated through April 30, 2025. The search strategy included decision support systems (BMJ Best Practice), guideline networks (NICE, GIN), and major electronic databases (Cochrane Library, PubMed, Web of Science, CNKI, Wanfang Data). Tailored search strategies were applied to each database using a combination of MeSH terms and free-text keywords. An example of the search strategy used in PubMed, illustrating the combination of these concepts, is provided below:

#1 “Cognition Disorders” [MeSH]

#2 “Cognitive dysfunction*” [Title/Abstract] OR “Cognitive disorder*” [Title/Abstract] OR “Cognitive impairment*” [Title/Abstract] OR “Mild cognitive impairment*” [Title/Abstract] OR “Cognitive decline*” [Title/Abstract] OR “Mental deterioration*” [Title/Abstract] OR “MCI” [Title/Abstract] OR “amnestic MCI” [Title/Abstract] OR “non-amnestic MCI” [Title/Abstract] OR “Preclinical Alzheimer*” [Title/Abstract] OR “Cognitive complaint*” [Title/Abstract]

#3 #1 OR #2

#4 “Exercise Therapy” [MeSH]

#5 “multicomponent training” [Title/Abstract] OR “Multicomponent exercise” [Title/Abstract] OR “Combined training” [Title/Abstract] OR “Mixed exercise” [Title/Abstract] OR “Integrated exercise” [Title/Abstract] OR “Multimodal exercise*” [Title/Abstract] OR “Concurrent training” [Title/Abstract] OR “Dual-task training” [Title/Abstract])

#6 #4 OR #5

#7 “Aged” [Mesh] OR “Aged, 80 and over” [Mesh] OR “Frail older adult” [Mesh] OR “older adult” [Title/Abstract] OR “Senior citizen*” [Title/Abstract] OR “Geriatric*” [Title/Abstract] OR “Older adult*” [Title/Abstract]

#8 “Systematic review” [Title/Abstract] OR “Meta-analysis” [Title/Abstract] OR “Evidence summar*” [Title/Abstract] OR “Consensus” [Title/Abstract]) OR “Review, systematic” [MeSH] OR “Practice Guideline” [MeSH]

#9 #3 AND #6 AND #7 AND #8

### 2.3 Source and basis of evidence

This evidence synthesis primarily integrates existing secondary research (such as systematic reviews, guidelines, and meta-analyses) to provide a comprehensive overview. It should be noted that the conclusions of this study are derived from these secondary sources; therefore, their robustness is directly constrained by the methodological quality of the cited reviews. For instance, perspectives on strength training are based on the review by Ahmadpoor and Bragazzi (21), while the discussion on exercise and psychological factors refers to the model proposed by Bahrami and Cranney (22). Readers should be aware that any limitations present in the original studies may influence the conclusions of this evidence synthesis as they cascade through the secondary literature.

### 2.4 Inclusion and exclusion criteria

Inclusion Criteria: Clinical guidelines, expert consensus, systematic reviews, evidence summaries, and meta-analyses related to multicomponent training (MT) in older adult individuals with mild cognitive impairment (MCI); Publications available in Chinese or English; The latest version of the literature if updates are available.

Exclusion Criteria: Studies involving participants under 60 years of age or those without a clearly defined MCI population; Studies that combine dementia populations without independently reported MCI subgroup data; Interventions lacking at least two core components: aerobic exercise, strength training, balance exercises, or flexibility; Literature without cognitive function outcome measures; Duplicate publications, draft guidelines, translations, or commentaries; Articles with missing full texts or critical information.

### 2.5 Literature quality assessment

The quality assessment was conducted by a team of four graduate nursing students, trained in Cochrane methods, and one evidence-based nursing expert. Following calibration exercises, inter-rater reliability was confirmed (ICC ≥ 0.9; Kappa ≥ 0.8). All literature was independently evaluated by two researchers, with a third arbitrator resolving any discrepancies. Specific assessment tools were applied as follows: Guidelines were evaluated using the AGREE II tool (23) and classified as: A (all six domains ≥60%), B (3–5 domains ≥60%), or C (fewer than three domains ≥60%). Systematic reviews/meta-analyses were assessed using AMSTAR 2 (24), with confidence levels (high, moderate, low, very low) determined based on critical domains such as protocol registration and comprehensive searching. Expert consensus documents were appraised using the JBI tool (2016) (25). Evidence summaries meeting the criteria for clinical relevance and sourcing transparency were designated as “lead documents.” All original studies were systematically traced and integrated with directly searched literature into a unified database for quality assessment. In total, 16 core references were included, comprising 3 guidelines, 1 expert consensus, 8 systematic reviews, and 4 meta-analyses. Conflicting findings were resolved by prioritizing higher-quality and more recent evidence.

### 2.6 Evidence extraction and synthesis

Evidence extraction was independently performed by two trained graduate students following a standardized protocol, with a focus on predefined domains such as intervention methods and program duration. During the synthesis process, the original wording of key findings was preserved to avoid misinterpretation. Similar evidence was thematically consolidated into structured recommendations, and complex multicomponent findings were deconstructed. To ensure a transparent and credible link between the critical appraisal of individual studies and the final evidence grading, the synthesis process explicitly incorporated the results of the quality assessments. In cases of conflicting findings, resolutions were prioritized based on the hierarchical level of evidence, methodological rigor (as assessed through the appraisal tools outlined in Section 2.4), and publication date. The Evidence Pre-grading System (2016) (26) was then applied to classify the evidence into Levels 1–4, primarily based on study design, but also moderated by the risk of bias and methodological limitations identified during the quality appraisal phase. Finally, recommendations were graded using the FAME framework: Grade A (derived from Level 1/2 evidence with high feasibility/applicability) and Grade B (from Level 3/4 evidence, or from Level 1/2 evidence with low feasibility or multiple low FAME ratings).

## 3 Results

### 3.1 Literature search results

**Figure 1:**
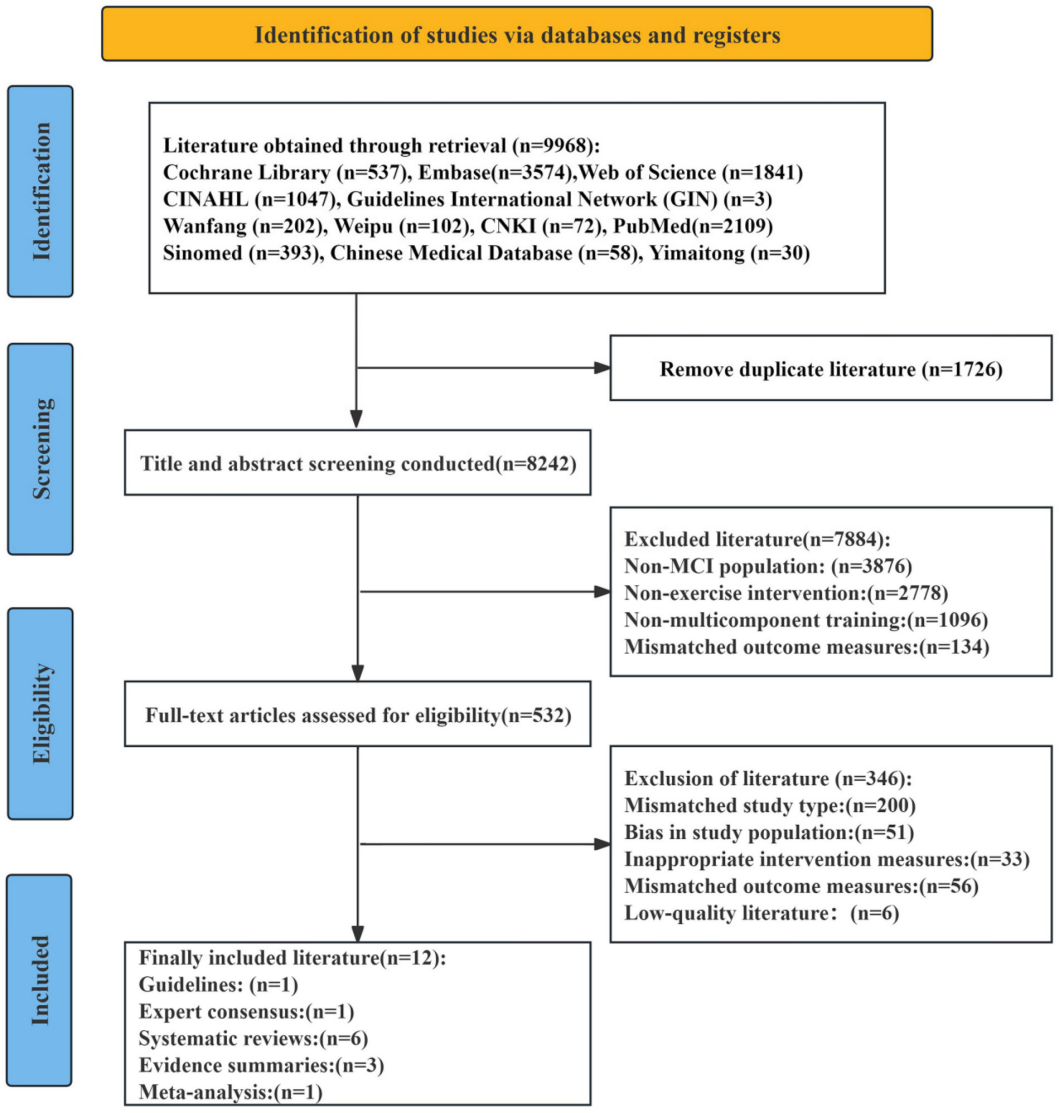
PRISMA flowchart of paper selection.

The initial database search identified 9,968 relevant articles. After removing 1,726 duplicates, 8,242 records remained. Title and abstract screening excluded 7,884 articles that did not meet the inclusion criteria. Full-text review and secondary screening of the remaining 358 articles led to the exclusion of 346 studies for the following reasons: 200 due to mismatched study types, 51 due to inappropriate study populations, 33 due to unsuitable intervention measures, 56 due to irrelevant outcome measures, and 6 due to low methodological quality. Ultimately, 12 articles were included in the final analysis. These consisted of one guideline, one expert consensus, six systematic reviews, three evidence summaries, and one meta-analysis. The screening process is depicted in Figure 1, and the basic characteristics of the included studies are summarized in Table 1.

**Table 1:** Basic characteristics of the included literature.

Author	Source of Literature	Type of Literature	Publication Year	Literature Topic	Primary Issues Identified
China Geriatric Nursing Alliance et al. (27)	Yimaitong	Guideline	2023	Exercise Intervention Guidelines for Older Adult with Cognitive Decline	1. Evidence quality is relatively low, with some studies having small sample sizes and inconsistent intervention measures; 2. Individualized guidance is insufficient, lacking detailed exercise plans tailored for patients with severe cognitive impairment
Liu Chang et al. (37)	Wanfang	Evidence summary	2023	Best Evidence Summary of Exercise Intervention for MCI Patients	1. The lack of original research targeting specific populations and intervention methods may affect the applicability of the recommendations
Wang Haiyan et al. (38)	Weipu	Evidence summary	2022	Best Evidence for Exercise Intervention in Older Adult MCI Patients: Modality Combination, Dosage parameters, and Safety Management	1. Evidence primarily stems from systematic reviews and expert consensus, lacking independent high-quality original research data. 2. Failure to account for patient individuality, absence of personalized interventions, and overemphasis on universal exercise regimens
Zhang Hongzhen et al. (39)	Wanfang	Evidence summary	2023	Best Evidence Summary of Exercise Intervention for Community MCI Patients	1. Evidence primarily stems from systematic reviews and expert consensus, lacking independent high-quality original research data. 2. There is insufficient data supporting the long-term effects of exercise interventions or sustained improvements in cognitive function. 3. Personalized exercise programs tailored to MCI patients with varying cognitive levels have not been established
Huang et al. (28)	Pubmed	Systematic review	2022	Comparsion of the Efficacy of various Exercise Interventions on Cognitive Function in MCI or Dementia Patients	1. The included RCTs exhibited considerable heterogeneity. 2. Reporting bias and selective reporting were present in this study, potentially affecting the comprehensiveness of the results
Venegas-Sanabria et al. (29)	Pubmed	Systematic review	2022	The Effect of Multicomponent training on Cognitive Impairment Patients	1. The findings exhibit significant heterogeneity, potentially undermining the reliability of conclusions. 2. The specific effects of different exercise combinations within multi-component interventions on cognitive function remain unclear, with no definitive conclusion on optimal intervention combinations. 3. Despite using the Cochrane risk of bias tool, risk of bias persists, affecting the accuracy of study results. 4. Inclusion was limited to English and Spanish language studies, compromising the comprehensiveness of findings
Yan et al. (30)	Web of science	Systematic review	2025	The Impact of Multicomponent training on Cognitive, Physical Function, and Daily Activities in Older Adult with Dementia or Mild Cognitive Impairment	1. The heterogeneity in intervention outcomes stems from the inclusion of multiple studies with differing intervention protocols. 2. There is a risk of publication bias. 3. Long-term follow-up studies on the effects of exercise interventions are lacking
Li et al. (31)	CINAHL	Systematic review	2024	The most suitable exercise strategies for patients with cognitive impairment and dementia	1. The optimal frequency and duration of exercise interventions remain unclear. 2. Issues related to blinding of researchers and evaluators may compromise the impartiality of study findings. 3. The current volume of research on multicomponent exercise programs is significantly lower than that on aerobic exercise and resistance training; further validation of MT efficacy is needed. 4. Detailed discussions on designing individualized exercise protocols for different types of MCI patients are lacking
Yu et al. (32)	Embase	Systematic review	2024	The best type and amount of exercise for improving cognitive function in patients with MCI	1. The included studies varied in intervention protocols (e.g., intensity, frequency, duration), resulting in heterogeneity in intervention effects. 2. Some included studies had small sample sizes, posing a risk of bias. 3. For multi-component exercise programs, specific proportions and intensities for each exercise component were lacking
Jia et al. (33)	Embase	Systematic review	2025	The effects of exercise intervention on older adult patients with MCI	1. The research findings exhibit high heterogeneity. 2. The lack of long-term follow-up on cognitive function prevents assessment of the intervention's sustainability
Liu et al. (34)	Pubmed	Meta-analysis	2025	The impact of multicomponent training on cognitive function in older adults with cognitive impairment	1. Despite employing a random-effects model for analysis, significant variations in intervention type, frequency, and duration across studies resulted in high heterogeneity in intervention outcomes. 2. Differences in intervention duration make it difficult to compare their respective effects
Cai et al. (36)	CNKI	Expert consensus	2021	Expert consensus on the dose-response relationship between physical exercise and the delay of cognitive decline in older adults	1. The literature primarily relies on moderate-quality RCT studies, and some studies have small sample sizes, which may influence the results. 2. While recommended exercise doses are provided for different activities, personalized intervention guidance is lacking

### 3.2 Literature quality evaluation results

#### 3.2.1 Guidelines

One guideline (27), sourced from the Medline database, was included. The results of the guideline quality evaluation are presented in Table 2.

**Table 2:** Quality evaluation results of the guidelines (*n* = 1).

Included Literature	Standardized percentage in each domain (%)						≥60%	ICC(2, k)	Overall quality (grade)
	Scope and objectives	Involved personnel	Rigor of guideline development	Clarity of guideline presentation	Applicability of the guideline	Independence in guideline drafting	Number of domains		
China Geriatric Nursing Alliance et al. (27)	97.22%	88.87%	79.18%	87.5%	30.21%	72.91%	5	0.89 (0.82-0.94)	B

#### 3.2.2 Systematic reviews and meta-analyses

A total of six systematic reviews were incorporated (28–33), with two retrieved from PubMed (28, 29), two from Embase (32, 33), one from CINAHL (31), and one from Web of Science (30). Overall, the quality of these reviews was relatively high, and all met the eligibility criteria for inclusion. Three meta-analyses were also identified: two from PubMed (17, 34) and one from Web of Science (35). Quality was assessed according to the seven key criteria established by the AMSTAR 2 team (criteria 2, 4, 7, 9, 11, 13, and 15). Based on this evaluation, the articles by Borges-Machado et al. (17) and Han et al. (35) were rated as “low” quality and excluded from further analysis. In contrast, the study by Liu et al. (34) demonstrated superior methodological quality and was retained. The detailed quality assessment results for systematic reviews and meta-analyses are shown in Table 3.

**Table 3:** Quality assessment results of systematic reviews and meta-analyses (*n* = 9).

Included studies	①	②	③	④	⑤	⑥	⑦	⑧	⑨	⑩	⑪	⑫	⑬	⑭	⑮	⑯
Huang et al. (28)	Y	Y	Y	Y	Y	Y	P	Y	Y	P	Y	Y	Y	Y	Y	Y
Venegas-Sanabria et al. (29)	Y	Y	Y	Y	Y	Y	Y	Y	Y	P	Y	Y	P	Y	Y	Y
Yan et al. (30)	Y	Y	Y	Y	Y	Y	Y	Y	Y	Y	Y	Y	P	Y	Y	Y
Li et al. (31)	Y	Y	Y	Y	Y	Y	Y	Y	Y	Y	Y	Y	P	Y	Y	Y
Yu et al. (32)	Y	Y	Y	Y	Y	Y	Y	Y	Y	Y	Y	P	Y	P	Y	Y
Jia et al. (33)	Y	Y	Y	Y	Y	Y	Y	Y	Y	Y	Y	P	Y	P	Y	Y
Liu et al. (34)	Y	Y	Y	P	Y	Y	P	Y	Y	N	Y	Y	Y	Y	Y	Y
Borges-Machado et al. (17)	Y	Y	Y	P	Y	N	Y	Y	Y	N	Y	N	Y	Y	Y	Y
Han at al. (35)	Y	Y	Y	P	Y	Y	N	Y	Y	N	Y	N	Y	Y	N	Y

Y indicates “Yes”, N indicates “No”, and P indicates “Partially Yes”. (1) Do the research question and inclusion criteria encompass all elements of PICO? (2) Was the methodology for the systematic review determined prior to its implementation, and were any discrepancies with the protocol reported? (3) Did the authors provide a rationale for selecting the type of study design included in the systematic review? (4) Did the authors employ a comprehensive literature search strategy? (5) Were two individual involved in the independent screening of the literature? (6) Were two individual involved in the independent extraction of data? (7) Was a list of excluded literature, along with the reasons for exclusion, provided? (8) Did the authors provide a detailed description of the basic characteristics of the included? (9) Did the authors employ appropriate tools to assess the risk of bias in the included studies? (10) Did the authors disclose the funding sources for the studies included in the systematic review? (11) If a meta-analysis was conducted, did the authors employ appropriate statistical methods for the results synthesis of results? (12) If a meta-analysis was conducted, did the authors consider the potential impact of risk of bias in the included studies on the meta-analysis or other evidence synthesis? (13) When interpreting or discussing the results of the systematic review, did the authors account for the risk of bias in the included studies? (14) Did the authors provide a satisfactory explanation or discussion regarding heterogeneity in the systematic review results? (15) If a quantitative synthesis was conducted, did the authors adequately investigate publication bias and address its potential impact on the study results? (16) Did the authors disclose any potential conflicts of interest, including any funding received for conducting the systematic review?

#### 3.2.3 Expert consensus

One expert consensus (36) was included. All entries were rated as “yes,” indicating high overall quality and eligibility for inclusion.

#### 3.2.4 Evidence summaries

Three evidence summaries (37–39), were incorporated. These summaries cited two guidelines (40, 41), one meta-analysis (42), and two systematic reviews (43, 44). Among the cited guidelines, one (40) was graded as B and the other (41) as A; both were considered high quality and included. The cited meta-analysis (42) lacked essential information for items 7, 13, and 15, resulting in a rating of “very low” quality; it was therefore excluded. The two systematic reviews (43, 44) received ratings of “no” for items 4 and 9, but “yes” for all remaining items. Their overall quality was judged to be high, and they were included.

#### 3.2.5 Evidence summary

The included evidence was subsequently synthesized, graded, and integrated across six key domains: (1) Principles for developing exercise prescriptions; (2) Intervention effects; (3) Exercise dosage and intensity; (4) Safety monitoring and risk management; (5) Methods for assessing intervention outcomes; (6) Implementation strategies and compliance management. This process yielded a total of 26 recommendation items, which are presented in Table 4.

**Table 4:** Best evidence for multicomponent exercise interventions targeting cognitive function in older adults with mild cognitive impairment.

Project	Best Evidence	Evidence Level
Principles for developing multicomponent training prescriptions	1. Early intervention is crucial for older adult individuals diagnosed with mild cognitive impairment (MCI) (39), emphasizing the reduction of sedentary behavior (avoiding prolonged bed rest or prolonged sitting) and the maintenance of baseline physical activity levels through daily tasks (such as household chores, walking to shop) as well as physical exercise (27, 32)	1a
	2. Social interaction should be integrate into exercise design, with tiered goals (short-term/long-term) being set and graded training being implemented, all with a focus on promoting behavioral change (36)	3b
	3. Before developing a plan, a comprehensive assessment should be conducted of the patient's physical fitness, cardiopulmonary function, the extent and scope of cognitive impairment, previous exercise habits and interests, and factors such as economic circumstances, family and accessible medical and social resources to develop a personalized plan (37)	3a
	4. The program should include aerobic exercise (such as brisk walking and swimming) combined with resistance training (such as weight training and resistance band exercises), balance exercises (such as standing on one leg), and flexibility training to significantly improve the overall cognitive abilities of MCI patient (29)	1a
	5. MT enhances cerebral blood flow, promotes BDNF release, and improves neural synaptic plasticity, resulting in a synergistic effect that surpasses the benefits exercise alone (29, 33)	1a
Intervention effects	6. MT resulted in significant improvements in the overall cognitive function and executive function of MCI patients (28, 32)	1a
	7. MT improves both physical flexibility, muscle strength, agility, and ADL independence (30)	1a
	8. MT improves both frailty and cognitive function (29)	1a
Exercise dosage and intensity	9. (a) Patients with mild cognitive impairment (MCI) should engage in MT 3–4 times per week, with each session lasting between 30 and 60 min (32, 39) (b) Patients with dementia or a tendency toward dementia should combine MT with aerobic training 2–3 times per week, with each session lasting for approximately 60 min (34)	1a 1a
	10. The total exercise volume should accumulate ≥150 min per week (27, 39)	1a
	11. Intensity should be primarily of moderate intensity (44), with exercise intensity reaching 60% or higher of maximum oxygen consumption or maximum heart rate, or assessed as “feeling slightly exerted” (RPE >13) using the Rating of Perceived Exertion (RPE) scale (36)	1a
	12. Exercise duration: Short-term (12–24 weeks) is aimed at improve specific cognitive domains (34, 36), while long-term (≥6 months) focuses on slowing cognitive decline and consolidating gains, and balancing safety and sustainability (42)	1a
Safety monitoring and risk management	13. A physical activity-based exercise risk assessment model should be used to identify potential cardiovascular events (e.g., angina, myocardial infarction) and exercise-related injuries (41)	1b
	14. During exercise, professionals or trained caregivers should continuously monitor patients’ heart rate, RPE (assessable via the Borg scale or Bruce modified protocol), breathing, sweating, and subjective feelings, monitoring for abnormal symptoms to ensure the safety of exercise intensity (27, 36)	1b
	15. Before exercise, the smoothness of the exercise venue's surface, traffic hazards, and social environmental safety should be assessed. It is important to prioritize familiar and convenient locations such as home or community centers (37, 39)	2a
	16. High-intensity exercise should be preceded by a proper warm-up, followed by post-exercise stretching and relaxation training to reduce the risk of injury. One-on-one guidance is required to prevent overexertion during a single session, with individual tolerance as the core principle. Exercise should be immediately stopped upon the onset of discomfort (27, 37)	1b
	17. Patients with chronic diseases should consult medical personnel before exercising and follow a conservative exercise regimen to ensure safety (39)	3a
	18. Patients with early- to mid-stage MCI should participate in coordination and balance training under safe conditions (38)	2a
Outcome assessment methods	19. Neuropsychological tests (e.g., MoCA, MMSE) should be used regularly to assess overall cognitive function in older adult MCI patients (39)	3a
	20. Conduct specialized assessments of different cognitive domains, including memory, executive function, and language function, to analyze the specific efficacy of various types of exercise in MT, focusing on expanding language function assessments to comprehensively reflect the dimensions of cognitive improvement (37)	3a
	21. Objective biomarker tests include cerebrospinal fluid markers, such as tau protein and Aβ1-42, plasma BDNF, and electroencephalogram (EEG) tests (36)	1b
Strategies for implementation and adherence management	22. Active participation of family caregivers in exercise training may enhance patients’ confidence in engaging in exercise (37)	3a
	23. Adherence can be improved through SMART goal setting, motivational interviewing, regular reminders, and dynamic adjustment of the program (34)	1a
	24. Prioritize exercise methods that do not require specific equipment to minimize barriers related to equipment availability. In developed areas, introduce technologies such as virtual reality to increase the enjoyment of exercise and enhance patient participation (37)	3a

## 4 Discussion

### 4.1 Scientific development of exercise prescriptions

Developing scientifically sound and feasible exercise prescriptions is crucial to maximizing the benefits of physical activity for patients with mild cognitive impairment (MCI). Evidence from this review (1–3) highlights the importance of tailoring exercise prescriptions based on comprehensive assessments of both internal and external factors. Internally, this includes evaluations of physical fitness, cardiorespiratory function, and the severity of cognitive impairment. Externally, factors such as prior exercise habits, interests, financial resources, family support, and access to community healthcare must also be considered. This comprehensive evaluation ensures both the safety of the intervention and the sustainability of adherence. Evidence 1 emphasizes the importance of early intervention and reducing sedentary behavior, which aligns with current strategies aimed at slowing cognitive decline (45). For individuals with limited physical capacity, even minimal physical activity is preferable to complete inactivity (46). Initiating light daily activities—such as household chores or walking short distances—can gradually increase intensity, ensuring both safety and effectiveness (47). Evidence 2 focuses on strategies to support long-term adherence. Embedding exercise within social contexts facilitates interaction, which not only enhances motivation but also provides cognitive stimulation. The use of stepwise goals, grounded in behavioral change theory (48), further reinforces sustainable exercise habits (39). Evidence 3 expands the scope of individualization beyond physiological factors. Elements such as family caregiving capacity and community infrastructure should be integrated into the prescription process. For individuals with financial constraints, equipment-free resistance training provides a cost-effective alternative. For those living alone, supervised community sessions with peer or volunteer involvement enhance both safety and feasibility. Collectively, these findings underscore the need for structured, individualized exercise prescriptions that are grounded in both scientific evidence and practical considerations.

### 4.2 Core value of multicomponent training: from single intervention to synergistic enhancement

Traditionally, single-component exercises were considered more appropriate for individuals with cognitive impairments due to their simplicit y (9). However, the present review (4, 5) indicates that most patients with MCI retain sufficient cognitive function to benefit from MT, which offers greater cognitive improvements than single-component interventions. Liu (49) found that MT has a more pronounced effect on MCI interventions. MT incorporates aerobic, resistance, balance, and flexibility training modalities. Aerobic exercise improves cardiorespiratory fitness and cognitive function, resistance training enhances muscle strength and supports activities of daily living, while balance and coordination exercises improve daily functioning and reduce fall risk. By capitalizing on the benefits of these diverse exercise modalities, MT significantly enhances both cognitive function and physical fitness in MCI patients. These combined mechanisms yield a significantly larger effect size for overall cognitive improvement compared to single-component exercises, consistent with findings by Huang (28).

A meta-analysis by Venegas-Sanabria (29) indicates that multi-component exercise programs can significantly improve overall cognitive function in MCI patients, but only when they include aerobic training. Aerobic exercise provides the physiological foundation for the synergistic effects of resistance and balance training by enhancing neurotrophic factor expression and promoting cerebral blood flow, among other mechanisms. Animal studies by Lan (50) suggest that combining aerobic exercise with strength training may negate the cognitive-enhancing effects of aerobic exercise. Furthermore, the intensity of strength training plays a crucial role in the combined effects of aerobic and strength training. Notably, the use of an MT program alone produces more pronounced effects on cognitive function enhancement in MCI patients. This suggests that incorporating MT into cognitive training for MCI patients may lead to superior outcomes.

However, findings by Tseng (51) diverge from this consensus, suggesting that single-component interventions may be more suitable for individuals with moderate-to-severe cognitive impairment. Three factors may explain this discrepancy: First, the complexity of MT increases the cognitive load for patients, potentially overshadowing the specific effects of certain exercise types for those with more severe cognitive impairment. Second, performing physical exercises and cognitive tasks simultaneously may lead to decreased task performance. For patients with severe MCI, single-task exercises may promote better concentration. Third, patients with severe MCI are at high risk of falls; high-intensity training may counteract the cognitive benefits of aerobic exercise, whereas single-task exercises allow for easier control of exercise intensity, reducing safety risks.

Future research should further investigate the complexity of MT, examine the specific effects of individual exercise components on cognitive function in MCI patients, and assess outcomes at various exercise intensities. For MCI patients with relatively preserved cognitive function, MT may be the preferred intervention. In contrast, patients with more severe cognitive impairment, who struggle with complex movements, could first develop foundational motor skills through single-modality exercises before gradually transitioning to MT. This personalized approach aims to optimize intervention outcomes and establish a refined, stratified management strategy for older adult MCI patients with varying cognitive levels.

### 4.3 Precision practice of exercise dosage and intensity

The precise control of exercise interventions is crucial for ensuring the efficacy of MT in patients with MCI. This requires clear definitions and scientific control of exercise dosage and intensity. However, considerable heterogeneity exists in the exercise frequencies that affect cognitive function. A meta-analysis by Afanador-Restrepo (52) indicates that, for older adults with cognitive impairment, exercising at lower frequencies (≤3 times per week) and shorter durations (approximately 60 min) results in more pronounced improvements in cognitive function. Similarly, a meta-analysis by Luo (53) confirmed that MT programs exceeding 120 min per week significantly enhance cognitive function and quality of life while reducing depressive symptoms in cognitively impaired older adults.

High-frequency, long-duration exercise helps patients achieve greater cognitive improvement, while low-frequency, short-duration exercise is more conducive to maintaining adherence to the exercise regimen (54). Although no definitive evidence currently identifies modifiable factors or barriers affecting exercise adherence in older adult MCI patients, shorter treatment durations may enhance sustained patient engagement (55). Given the heterogeneity of existing research and the lack of evidence for personalized exercise interventions, a synthesis of 9–12 evidence sources recommends the following exercise dosage and intensity for MT to improve cognitive function in MCI patients: 3–4 sessions per week, each lasting 30–60 min, accumulating to a total weekly duration of ≥150 min of moderate-intensity physical activity. Future validation may be achieved through large-scale randomized controlled trials or cohort studies with substantial sample sizes.

Regarding exercise intensity, Evidence 11 and the subgroup analysis by Biazus-Sehn (56) explicitly recommend moderate intensity, defined as achieving 60% of heart rate reserve (HRmax) or a rated perceived exertion (RPE) >13 in MCI patients. In clinical practice, healthcare providers must thoroughly assess patients' baseline physical fitness, cognitive status, and adherence to establish individualized exercise prescriptions.

Evidence 12 highlights the association between intervention duration and outcomes. A meta-analysis by Liu (34) indicates that MT interventions lasting 12–24 weeks yield the most significant effects, rapidly activating neuroplasticity and improving specific cognitive domains—particularly executive function, visual memory, and verbal memory. For patients newly diagnosed with MCI or in the early stages of cognitive decline, early intervention offers the greatest potential for reversal. Long-term maintenance of MT for ≥6 months results in stable gains in language abilities (42), making it suitable for patients who have completed short-term interventions and reached a stable phase on the Montreal Cognitive Assessment (MoCA).

For patients with dementia, the primary goal is to delay functional decline. Related studies suggest that long-term intervention does not guarantee sustained cognitive improvement (57). Regular adjustments to exercise protocols are necessary to prevent physical adaptation and decline in patients. Therefore, MT should be individualized based on improvements in patients' physical fitness, functional status, or disease treatment outcomes. This approach considers the relationship between exercise dosage, intensity, and cognitive function improvement to maximize the delay of cognitive decline in older adult MCI patients.

### 4.4 Safety monitoring and risk management as prerequisites for program implementation

Pre-exercise risk assessment is essential to ensure patient safety and should include evaluations of cardiovascular, metabolic, musculoskeletal, and cognitive conditions. The Vivi frail protocol (58) is recommended to identify contraindications, excluding severe conditions such as respiratory failure, and to guide safe adaptations for relative risks. Functional tests, such as the Short Physical Performance Battery (SPPB), assess balance, gait speed, and strength, providing critical data for risk stratification and intensity planning (59). Interventions should not commence without these assessments. Real-time monitoring is particularly important in home settings, where post-discharge patients often lack supervision, thereby increasing the risk of adverse events (60). The use of wearable technologies to monitor heart rate, SpO2, and perceived exertion can extend professional oversight into patients' homes. Source 15 recommends thorough evaluation of exercise environments, with a preference for familiar and safe locations, such as homes or community centers. This is particularly crucial for MCI patients who are prone to spatial disorientation. Source 16 provides safety guidance for high-intensity interventions, advocating for a dynamic balance between intensity and safety. Sources 17 and 18 emphasize the need for individualized modifications based on patient-specific risk profiles to ensure optimal benefit while maintaining safety.

### 4.5 Multi-dimensional integration of effect monitoring

Effective monitoring of MT outcomes requires a multi-dimensional approach, extending beyond any single measure. Evidence from sources 20–23 supports the integration of cognitive, physiological, and functional indicators into a dynamic data chain to guide personalized interventions. Cognitive assessment should combine the Mini-Mental State Examination (MMSE) and the Montreal Cognitive Assessment (MoCA). The MMSE is effective for evaluating orientation and language (61, 62), but lacks sensitivity for detecting early-stage deficits. In contrast, the MoCA captures executive and visuospatial functions (63), providing a more comprehensive assessment of cognitive domains (64). However, both scales are subjective and can be influenced by patient mood or cooperation. Electroencephalography (EEG) offers an objective alternative, with markers such as P300 latency reflecting early cognitive changes (65). When used alongside the MMSE and MoCA, EEG helps validate improvements and minimizes the bias inherent in relying on a single measure. Despite its potential, EEG is currently confined to research settings due to high costs and the absence of standardized protocols. Consequently, standard cognitive scales remain the primary tools in clinical practice. Future research should focus on developing reference standards for EEG-based indicators, such as P300 latency, under various exercise conditions.

### 4.6 Multi-dimensional improvement of implementation strategies and compliance management

This study identifies the primary barriers to low exercise adherence among patients with MCI, including a lack of external support systems, reduced motivation due to depressive mood, and variability in application scenarios. These factors directly impact the successful implementation of exercise programs. To overcome these challenges and improve adherence, comprehensive support measures are essential. Evidence from sources 24–25 emphasizes that emotional support from family caregivers plays a critical role in enhancing adherence. Therefore, clinical practice should integrate caregivers into collaborative systems to improve exercise adherence in older adult MCI patients. Training caregivers equips them with strategies to effectively motivate patients to exercise and offers guidance on providing exercise support tailored to the patients’ cognitive and emotional states.

Moreover, healthcare providers can enhance patients' intrinsic motivation and exercise adherence through psychological interventions, including the use of SMART goals and regular reminders. A meta-analysis by Noone (66) indicates that psychosocial interventions can effectively alleviate depressive or anxiety symptoms in individuals with dementia or MCI. Evidence 26 highlights management strategies for MT across different exercise settings. Research by Frändin (67) suggests that MT under professional supervision yields the most beneficial effects on cognitive function in older adults. In resource-limited settings, home-based exercise serves as a supplementary measure to professional oversight. Family members must collaborate effectively to ensure the safe and effective implementation of exercise programs for older adult MCI patients. The flexibility of the MT prescription allows for varied implementation methods and settings. With guidance and accompaniment from community healthcare providers and family members, older adult MCI patients can maintain motivation and improve adherence to exercise regimens (68).

Prioritizing equipment-free exercises can broaden accessibility by lowering environmental barriers. In regions with access to technology, virtual reality (VR) technology is increasingly being incorporated into rehabilitation care. Through scenario-based interactions, VR enhances training engagement and addresses the monotony of traditional intervention models (9). Virtual health interventions offer patients the ability to access healthcare services anytime and anywhere, thereby improving adherence and health outcomes. Additionally, these interventions provide potential for continuous monitoring and feedback, aiding in the optimization of interventions and the delivery of personalized care.

Compared with previous studies (30, 36), this research conducted an in-depth exploration of cognitive function improvement in MCI patients through comprehensive analysis of the dosage, intensity, and intervention patterns of MT. It provides more personalized exercise prescription recommendations, clarifies the comprehensive benefits of MT on cognition, physical fitness, and activities of daily living in older adult MCI patients, and offers more operational and practical solutions for the comprehensive health management of older adult MCI patients.

## 5 Limitations

This study is subject to several limitations. First, the evidence is primarily drawn from secondary sources, including systematic reviews and meta-analyses. The conclusions of these studies are dependent on the quality of the original research and may be influenced by publication bias, potentially undermining the accuracy of effect size estimates and obscuring methodological flaws within the primary studies. Second, the literature search was limited to publications in Chinese and English, which may have excluded significant evidence published in other languages, thus affecting the comprehensiveness of the evidence base. Third, the included evidence lacks sufficient detail on key aspects, such as the optimal ratio of MT components, advanced standards, and guidelines for individualized adjustments. This gap in reporting restricts the applicability and scalability of the evidence to clinical or community practice. Therefore, further high-quality research is needed to validate and refine these findings.

## 6 Conclusions

This study synthesizes the highest-quality evidence regarding the enhancement of cognitive function in older adult patients with MCI through MT. A total of 24 pieces of evidence were reviewed, including 20 Grade A and 4 Grade B recommendations. These findings offer evidence-based guidance for healthcare professionals to develop individualized MT programs within community and long-term care settings. However, it is crucial that these programs be adaptively tailored to the individual needs of patients, considering factors such as functional status, resource availability, and cultural preferences. Future research should focus on conducting high-quality randomized controlled trials (RCTs) to identify the optimal combinations and progression pathways of MT components. Additionally, studies should explore the integration of home monitoring technologies, ensuring that such interventions are feasible and effective in real-world settings.

## 7 Implications for practice

Healthcare clinicians are encouraged to use this synthesized evidence when formulating personalized multi-component exercise prescriptions for older adults with MCI. Clinical practice should adhere to the recommended principles, dosage parameters, and safety protocols to ensure interventions are evidence-based, systematically implemented, and rigorously monitored to effectively mitigate cognitive decline.

## Data Availability

The data analyzed in this study is subject to the following licenses/restrictions: the data that support the findings of this study are available from the corresponding author upon reasonable request. Requests to access these datasets should be directed to wyl3001@yeah.net.
